# Recombinant baculovirus expressing the FrC-OVA protein induces protective antitumor immunity in an EG7-OVA mouse model

**DOI:** 10.1186/s13036-019-0207-y

**Published:** 2019-10-22

**Authors:** Keigo Kondou, Tomoyuki Suzuki, Myint Oo Chang, Hiroshi Takaku

**Affiliations:** Department of Life and Environmental Sciences, Chiba Institute of Technology, 2-17-1 Tsudanuma, Narashino, Chiba, 275-0016 Japan

**Keywords:** Recombinant baculovirus, Wild-type baculovirus, Fragment C of tetanus toxin, Ovalbumin, T cells, Tumor immunity

## Abstract

**Background:**

The baculovirus (BV) *Autographa californica* multiple nuclear polyhedrosis virus has been used in numerous protein expression systems because of its ability to infect insect cells and serves as a useful vaccination vector with several benefits, such as its low clinical risks and posttranslational modification ability. We recently reported that dendritic cells (DCs) infected with BV stimulated antitumor immunity. The recombinant BV (rBV) also strongly stimulated peptide-specific T-cells and antitumor immunity. In this study, the stimulation of an immune response against EG7-OVA tumors in mice by a recombinant baculovirus-based combination vaccine expressing fragment C-ovalbumin (FrC-OVA-BV; rBV) was evaluated.

**Results:**

We constructed an rBV expressing fragment C (FrC) of tetanus toxin containing a promiscuous MHC II-binding sequence and a p30-ovalbumin (OVA) peptide that functions in the MHC I pathway. The results showed that rBV activated the CD8^+^ T-cell-mediated response much more efficiently than the wild-type BV (wtBV). Experiments with EG7-OVA tumor mouse models showed that rBV significantly decreased tumor volume and increased survival compared with those in the wild-type BV or FrC-OVA DNA vaccine groups. In addition, a significant antitumor effect of classic prophylactic or therapeutic vaccinations was observed for rBV against EG7-OVA-induced tumors compared with that in the controls.

**Conclusion:**

Our findings showed that FrC-OVA-BV (rBV) induced antitumor immunity, paving the way for its use in BV immunotherapy against malignancies.

## Background

The baculovirus (BV) system is used for the production of various vaccine candidates, inducing humoral and cell-mediated cross-immunity to viral infections [1–3]. We previously demonstrated that the wild-type (wt) BV *Autographa californica* multiple nuclear polyhedrosis virus (AcMNPV) or BV-infected dendritic cells (DCs) exert natural killer (NK) and CD8^+^ T cell-dependent antimetastatic effects on mice, but they are CD4^+^ T cell independent [4–7]. These antimetastatic effects involve BV directly activating NK cells by inducing the upregulation of NK cell effector function against the tumor in a Toll-like receptor 9 (TLR9)-dependent manner [8]. Additionally, BV has been shown to suppress liver injury and fibrosis in vivo through the induction of interferon (IFN) [9]. Molinari et al. [10] also reported that BV carrying ovalbumin (OVA) on the VP39 capsid protein induced antitumor immunity.

On the other hand, studies by several research groups have demonstrated that the high titer recombinant BV (rBV) antigen can induce specific antibodies [11–13]. The high-level transgene expression from rBV vectors is well suited for antitumor therapy and has been tested in animal tumor models [14–16].

Therefore, in the present study, an rBV-based combination vaccine was developed that expressed fragment C (FrC) of tetanus toxin containing a promiscuous MHC II-binding sequence [17] and a p30-OVA peptide that functions in the MHC I pathway [18], and its potential as an antitumor vaccine was evaluated.

## Results

### Preparation of BV expressing FrC-OVA

**Fig. 1:**
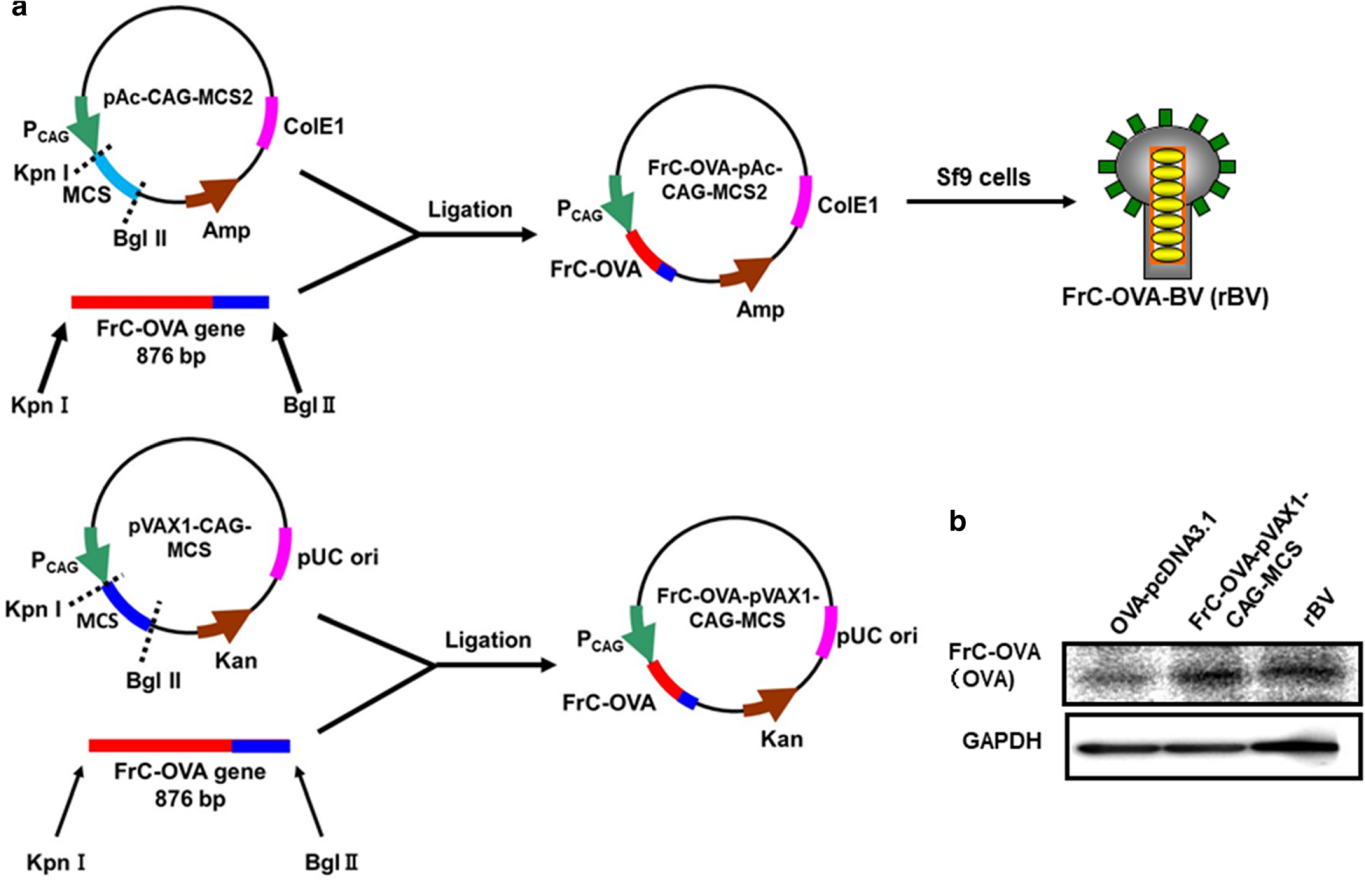
Construction of the BV transfer vector and FrC-OVA expression DNA vaccine. **a** Construction of the FrC-OVA-pAc-CAG-MCS2 and FrC-OVA-pVAX1-CAG-MCS plasmids containing the gene encoding the first domain of the FrC of tetanus toxin (TT865–1120). These plasmids were constructed to encode OVA 257–269 peptides fused directly to FrC. **b** Expression of the OVA protein in HEK-293 T cells infected with rBV or transfected with FrC-OVA-pVAX1-CAG-MCS. The cell extracts were separated by SDS-PAGE and analyzed by immunoblot using an anti-OVA antibody. Lane 1, transfected with OVA-pcDNA3.1 using FuGEN6; lane 2, transfected with FrC-OVA-pVAX1-CAG-MCS using FuGENE-6, lane 3, infected with rBV at an MOI of 100

The PCR products of OVA and FrC-DNA fragments were inserted between the *Kpn*I and *Bgl*II sites under the CAG promoter of pAc-CAG-MCS2 or pVAX1-CAG-MCS to construct the recombinant plasmids FrC-OVA-pAc-CAG-MCS2 and FrC-OVA-pVAX1-CAG-MCS, respectively (Fig. 1a). The insertion of FrC-OVA into plasmids was confirmed by RT-PCR analysis (Additional file 1: Figure S1). The production of FrC-OVA-BV (rBV) and wtBV is described in the Materials. High-titer viruses were obtained, ranging from 1 × 10^4^ to 1 × 10^9^ FFU/ml, and the structure of FrC-OVA on rBV-genomic DNA was confirmed by western blot analysis of rBV-infected HEK-293 T cells (Fig. 1b).

### IFN-γ response in mice injected with rBV

**Fig. 2:**
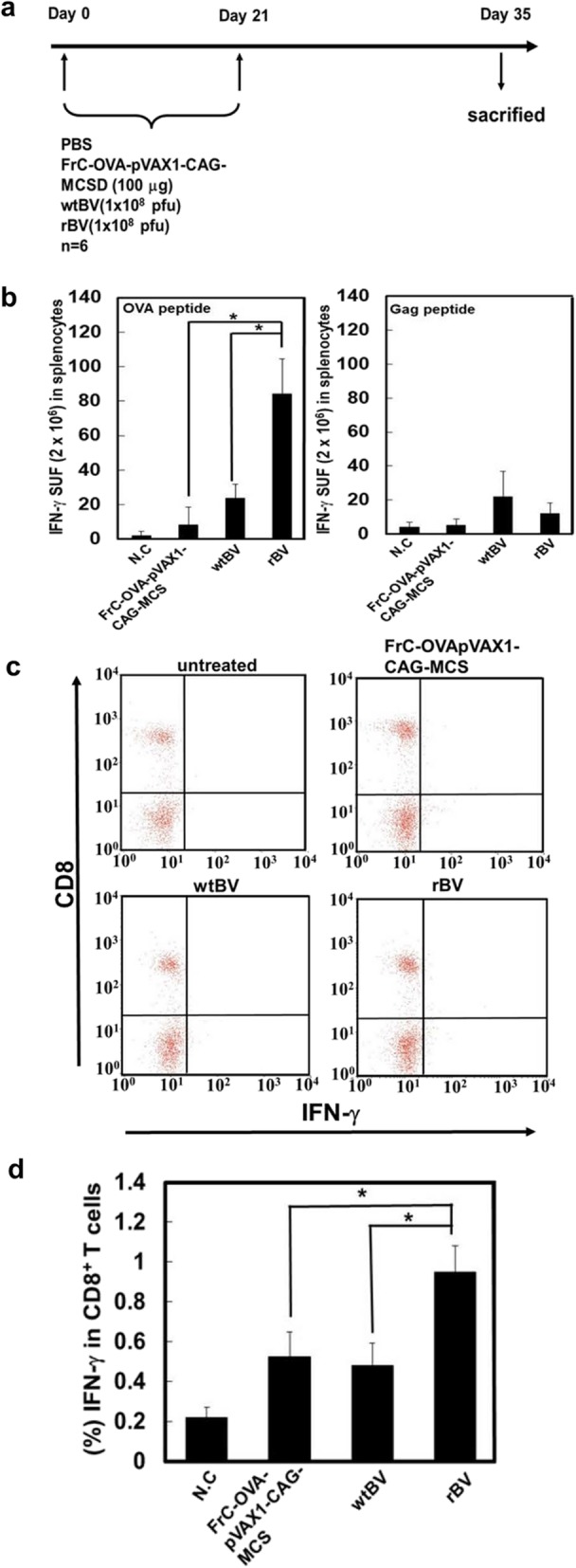
Vaccination induces OVA-specific IFN-γ-secreting spleen cells or CD8^+^ T cells in B6 mice. **a** Schematic of the experimental design of mouse immunization. Six-week-old B6 mice were vaccinated with FrC-OVA-pVAX1-GAG-MCS, wtBV, rBV or PBS on days 0 and 21 with the same vaccine via intramuscular injection. On day 35, the mice were sacrificed, and their spleens were isolated. **b** The IFN-γ contents in the supernatants of spleen cells from immunized mice were determined using IFN-γ ELISPOT analysis. Spleen cells were recovered and cultured for 24 h in the presence of OVA or HIV-1 Gag proteins. As a control, unstimulated spleen cells were cultured. **c** Intracellular staining of IFN-γ in splenocytes immunized with FrC-OVA-pVAX1-GAG-MCS, wtBV, rBV or PBS as indicated above. The spleen cells were incubated with the OVA peptide and brefeldin A for 4 h. The intracellular production of IFN-γ in the population of CD8^+^ T cells was then analyzed by flow cytometry. **d** Percentage of IFN-γ in CD8^**+**^ T cells. The results are representative of three independent experiments with six mice per group, and the error bars indicate the standard deviations of the mean values. ^*^P < 0.05 (Student’s t-test)

An rBV vaccine that expressed tetanus toxin FrC containing a promiscuous MHC II-binding sequence and a p30-OVA peptide that functions in the MHC I pathway was constructed. Because FrC-OVA-BV (rBV) is specific to the MHC I pathway, we evaluated its OVA-specific IFN-γ secretion in vivo*.* The OVA-specific IFN-γ-producing T-cells from splenocytes were analyzed using ELISPOT or CD8^+^ T-cell IFN-γ assays 35 days after the intramuscular injection of rBV, wtBV, FrC-OVA-pVAX1-CAG-MCS or PBS on days 0 and 21 in mice (Fig. 2a). As displayed in Fig. 2b, the restimulation of rBV-immunized spleen cells with the OVA peptide resulted in higher levels of OVA-specific IFN-γ compared with those in cells treated with wtBV, FrC-OVA-pVAX1-CAG-MCS or PBS. In the rBV-immunized spleen cells treated with the control peptide HIV-1 Gag, the level of OVA-specific IFN-γ was decreased to that observed in the wtBV control. On the other hand, as determined by the CD8^+^ T-cell IFN-γ assay, the rBV, wtBV and FrC-OVA-pVAX1-CAG-MCS groups showed higher levels of CD8^+^ T-cell IFN-γ than the PBS control group (Fig. 2c and d). These results suggest that rBV is more efficient at activating the CD8^+^ T-cell-mediated response than wtBV or FrC-OVA-pVAX1-CAG-MCS groups.

### Antitumor effects of rBV against EG7-OVA-induced tumors

**Fig. 3:**
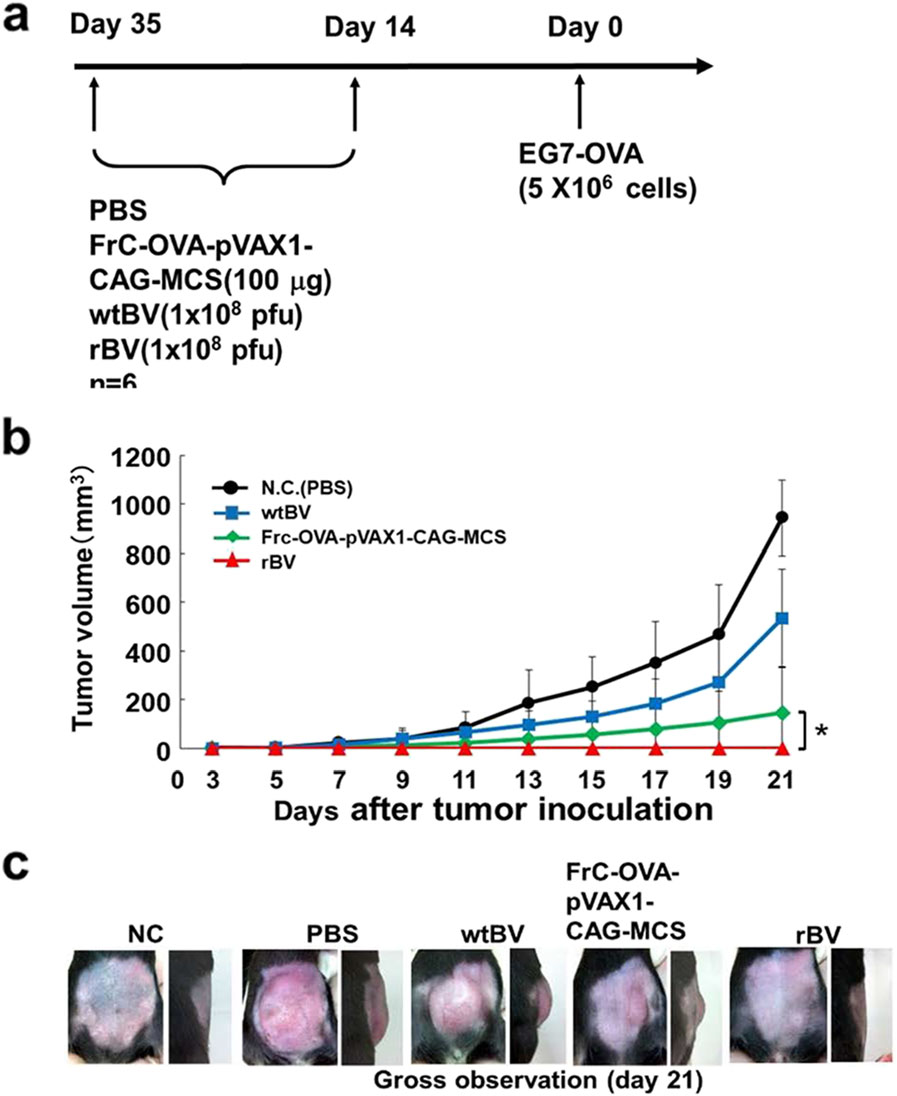
Effect of a classic prophylactic vaccination with rBV on EG7-OVA cells in mice. **a** Experimental dosing was used to assess the antitumor immunity conferred by rBV. B6 mice were immunized with a single intramuscular injection of FrC-OVA-pVAX1-CAG-MCS (100 μg), wtBV (1 × 10^8^ pfu), rBV (1 × 10^8^ pfu) or PBS. At 14 and 35 days, EG7-OVA cells (5 × 10^6^ cells/animal) were administered subcutaneously to the immunized mice. **b** The tumor volumes were measured every 2 days for 3 weeks. **c** Characterization of the established EG7-OVA tumor. Similar results were obtained in two independent experiments with 6 mice per group. The data are presented as the mean ± SD. ^*^P < 0.05

**Fig. 4:**
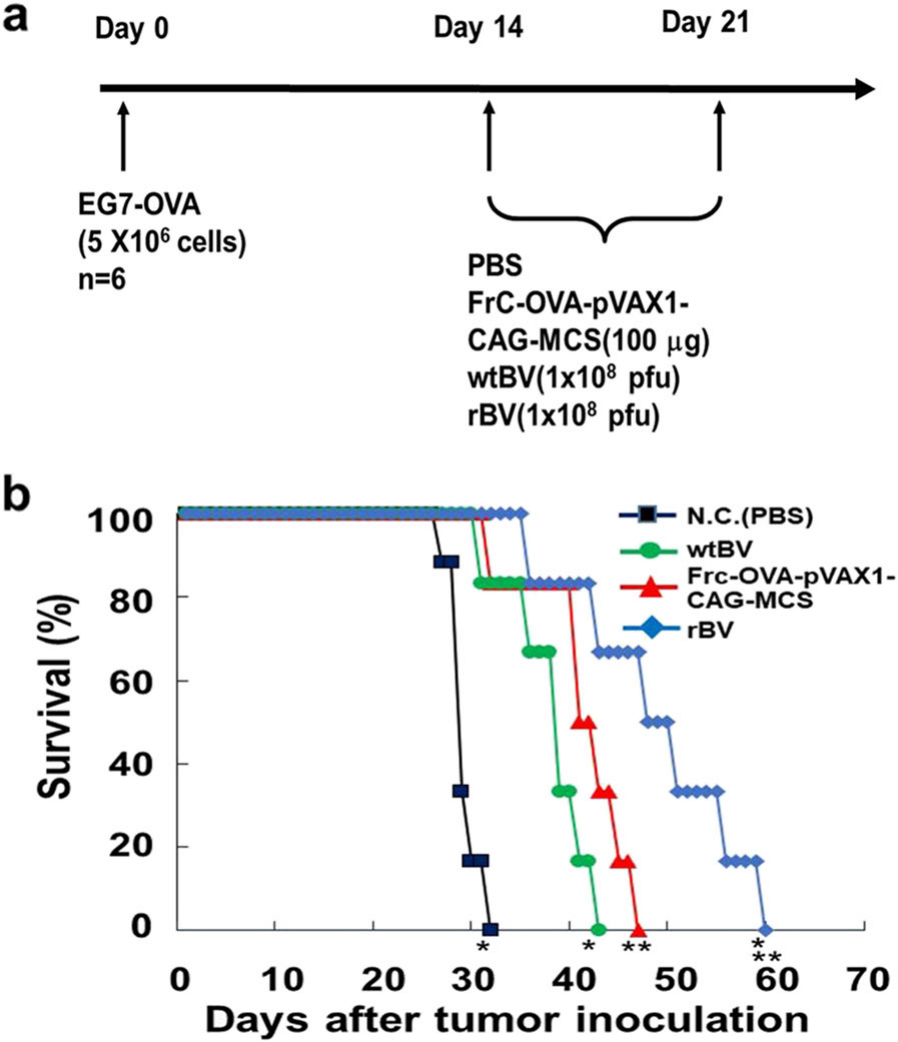
Therapeutic vaccination with rBV on EG7-OVA-induced tumors in mice. **a** Schematic of the experimental timeline. **b** Comparison of the antitumor immunity exhibited by rBV, FrC-OVA-pVAX1-CAG-MCS or wtBV in B6 mice. Mice were subcutaneously injected on day 0 with EG7-OVA cells (5 × 10^6^ cells/mouse). Survival rates of EG7-OVA-injected mice treated with rBV, FrC-OVA-pVAX1-CAG-MCS, wtBV or PBS on days 14 and 21. ^*^P < 0.01 compared with the negative control and wtBV groups. ^**^P < 0.05 compared with the FrC-OVA-pVAX1-CAG-MCS group. Similar results were obtained from two independent experiments with 6 mice per group

Experiments were performed to verify whether rBV could induce antitumor immunity against established subcutaneous tumors in mice. First, a classic prophylactic vaccination was examined. The experimental design is demonstrated in Fig. 3a. Mice were immunized with rBV, wtBV, FrC-OVA-pVAX1-CAG-MCS or PBS at 35 and 14 days prior to being inoculated with EG7-OVA cells. The growth of the tumors in the rBV group was inhibited compared with that observed in the PBS control group (no tumors developed), whereas the tumor growth in the wtBV or FrC-OVA-pVAX1-CAG-MCS groups did not differ from that in the controls until day 9, but inhibition was noted thereafter (Fig. 3b and c). To further determine the antitumor effects of rBV against EG7-OVA-induced growth, a therapeutic vaccination was performed (Fig. 4a). The survival rates of mice that were inoculated with EG7-OVA cells on day 0 followed by immunization with rBV, wtBV, FrC-OVA-pVAX1-CAG-MCS or PBS on days 14 and 21 were calculated. The survival times of the mice immunized with rBV were significantly longer than those of the mice inoculated with control wtBV or FrC-OVA-pVAX1-CAG-MCS (Fig. 4b). These results indicate that rBV-mediated protection against EG7-OVA-induced tumors may be a useful antitumor immunotherapy tool.

## Discussion

The viral vector vaccines established to date are human viral vectors that continue to give rise to problems associated with biosafety and toxicity. Furthermore, inactivated vaccine-mediated immunity is short-lived and predominantly humoral, with poor cell-mediated immunity. Among viral vectors, adenovirus and adeno-associated virus (AAV) have a much lower risk of insertional mutagenesis and have been tested for oncolytic virotherapy [19–21]. Adenoviral vectors will elicit antiviral immune responses following the first administration with a large dose of the vector because the virus is highly immunogenic [19–23]. This reaction might preclude further use of vectors or make subsequent use less effective. Recently, certain nonhuman viral vectors, including BV, have been explored as gene therapy vectors. Previous studies focused on the use of BVs as vaccines in gene therapy, as BVs do not replicate in mammalian cells and have low cytotoxicity and favorable biosafety features [24–28]. Kim et al. used BV as a delivery system to introduce telomerase reverse transcriptase (TERT) as a potential tumor-associated antigen for cancer immunotherapy [29]. In immunocompetent mice, BV-TERT induced IFN-γ T cells specific for TERT and NK cell activity in mouse splenocytes [29]. Since surface modification of the BV envelope by the vesicular stomatitis G (VSV-G) membrane protein improves BV transduction in vitro or in vivo, the display of the VSV G protein (VSVG) and heterologous peptide/protein via the GP64 anchor are the most widely adopted methods for enhancing the in vitro and in vivo gene transduction efficiency of recombinant baculoviruses [30–36]. Using this method, LyP-1, F3, and CGKRK tumor-homing peptides were originally identified by the in vivo screening of phage display libraries [37]. The fusion proteins were successfully incorporated into budded virions, which showed binding abilities to human breast carcinoma (MDA-MB-435) and hepatocarcinoma (HepG2) cells that were improved two- to fivefold. These fusion proteins inhibited virus biding and transduction by free soluble peptides. The soluble Lyp-1 peptide induces death in cultured cancer cells and inhibits tumor growth in mice implanted with xenograft tumors. The authors described that a BV expressing LyP-1 exerted an effect similar to that of soluble LyP-1 [38]. In addition to allowing efficient BV transduction, wtBV has been shown to immunostimulate the release of inflammatory cytokines, including INFs, TNF-α, IL1A, IL1B and IL6 in mammalian cells and confer protection from lethal virus infection in mice [39, 40]. Abe et al. reported that wtBV activates proinflammatory cytokines in peritoneal macrophage cells, splenic CD11c^+^ DCs, and a murine macrophage cell line through the TLR9/MyD88 pathway in which the BV genome induces the innate immune response [41]. Subsequently, our previous studies demonstrated that BV induces the functional maturation of human monocyte-derived DCs (HCDs) and the activation of human NK cells via BV-HCDs [6, 7]. Furthermore, we showed that BV directly actives NK cells via TLR9 [8]. BV-DCs might therefore be a useful immunotherapy tool for viral infections and malignancies, particularly if used in association with current virotherapies to achieve the most effective results. The host innate and acquired or adaptive immunity were also strongly induced by the rBV vectors.

In the present study, an anticancer combination therapy was investigated using an rBV-based combination vaccine expressing the FrC of the tetanus toxin and the OVA peptide (FrC-OVA), and its synergistic action as an antitumor vaccine was evaluated. For this investigation, FrC-OVA-BV (rBV), wtBV and FrC-OVA-pVAX1-CAG-MCS were constructed.

Using an EG7-OVA tumor mouse model, this study aimed to examine whether IFN-γ production by FrC-OVA-BV (rBV) was OVA-specific. IFN-γ release ELISPOT or CD8^+^ T-cell IFN-γ assays were used (Fig. 2a-d). In response to the OVA peptide, rBV exhibited significantly higher OVA-specific IFN-γ levels than wtBV or FrC-OVA-pVAX1-CAG-MCS groups. However, compared with that in the rBV-immunized spleen cells that were restimulated with the control HIV-1 gag peptide, the level of OVA-specific IFN-γ production was reduced to the level observed in the wtBV control. On the other hand, in the CD8^+^ T-cell IFN-γ assay, the rBV, wtBV, and FrC-OVA-pVAX1-CAG-MCS groups displayed higher levels of CD8^+^ T-cell IFN-γ than the PBS control group. In the present study, the question of whether rBV can induce the effects of antitumor immunity against EG7-OVA cells in mice was addressed. Classic prophylactic or therapeutic vaccinations were examined. These vaccinations resulted in the inhibition of tumor growth and an increased survival time in response to inoculation with rBV (Figs. 3b and c, and 4b). These results indicate that the antitumor effects of rBV against EG7-OVA-induced tumors are mediated by a specific anti-FrC-OVA immune response.

## Conclusion

The results of the present study revealed that an rBV-based combination vaccine expressing FrC-OVA sufficiently induced antitumor immunity in mice with established tumors and was more effective than wtBV. In addition, rBV against EG7-OVA showed a significant antitumor effect in classic prophylactic or therapeutic vaccinations. Furthermore, baculovirus has several attractive advantages, such as its good biosafety, large capacity for foreign genes, and better posttranslational modifications than those of other gene delivery vehicles. Therefore, rBV is potentially useful as an efficient antimetastatic agent and is expected to be advantageous in the future development of antitumor therapies.

## Materials

### Cell culture and reagents

Female C57BL/6 mice (B6) (6 weeks old) were purchased from Japan SLC, Inc. and maintained under humane and specific pathogen-free conditions according to the rules and regulations of the institutional committee of Chiba Institute of Technology, Narashino, Japan. *Spodoptera frugiperda* (Sf9) insect cells were cultured in Sf-900 II culture medium (Invitrogen; Thermo Fisher Scientific, Inc.). HEK-293 T cells were cultured in DMEM (Sigma-Aldrich; Merck KGaA) supplemented with 10% fetal bovine serum (FBS; Thermo Fisher Scientific, Inc.), 100 U/ml penicillin and 100 μg/ml streptomycin (both Sigma-Aldrich; Merck KGaA). EG7-OVA cells (EL4 derivative, ATCC® CRL-2113™, American Type Culture Collection) were maintained in complete RPMI 1640 medium (Gibco; Thermo Fisher Scientific, Inc.) supplemented with 10% heat-inactivated fetal calf serum, 2 mM L-glutamine, penicillin (0.1 U/ml) and streptomycin (0.1 mg/ml) and adjusted to contain 1.5 g/l sodium bicarbonate, 4.5 g/l glucose, 10 mM Hepes, 1 mM sodium pyruvate, 0.05 mM 2-mercaptoethanol and 0.4 mg/ml G418 at 37 °C in a 5% CO_2_ atmosphere.

### Plasmids

The DNA vaccine containing the gene encoding the first domain (p. DOM) was constructed by PCR amplification of the N-terminal domain sequence (TT865–1120) from p. FrC using the forward primer Kpn I-DOM-F (5′-CGGGGTACCGCCGCCACCATGGGTTGGAGCTGTATCAT-3′) and the reverse primer Bgl II-DOM-R containing the OVA peptide sequence (257–269) (5′-GAAGATCTTTAACTGGTCCATTCAGTCAGTTTTTCAAAGTTGATTATACTGTTACCCCAGAAGTCACGCA-3′) before cloning into pAc-CAG-MCS2. To generate pVAX1-CAG-MCS, the *MluI*/*Bam*HI-digested DNA fragment of pAc-CAG-MCS2 [42] was inserted into *MluI/Bam*HI-digested pVAX1-CMV-MCS. The PCR product (FrC-OVA) was inserted between the Kpn I and Bgl II sites under the CAG promoter of pAc-CAG-MCS2 or pVAX1-CAG-MCS. The PCR products of OVA and the Fc-DNA fragments were cloned into pcDNA3.1 to construct the recombinant OVA-pcDNA3.1 plasmids. The plasmids propagated in *Escherichia coli* were purified with the Qiagen Plasmid Mini kit (Qiagen GmbH) according to the manufacturer’s protocols. The insertion of FrC-OVA into plasmids was confirmed by RT-PCR analysis of total RNA isolated from rBV-infected HEK-293 T cells (Additional file 1: Figure S1) and sequence analysis.

### Preparation of BV

AcMNPV and rBV were propagated in Sf9 cells cultured in TMN-FH medium (BD Biosciences) containing 100 μg/ml kanamycin and 10% FBS. Ac/CAG-FrC-OVA and AcMNPV were purified as described previously [39, 43, 44]. The viral titers were determined by a plaque assay.

### Reverse transcription (RT)-PCR analysis

Total RNA was extracted from cells using the GenElute™ Mammalian Total RNA Miniprep kit (Sigma-Aldrich; Merck KGaA) according to the manufacturer’s protocols. cDNA was prepared using ReverTra Ace-α-™ (Toyobo Life Science). The PCRs for FrC-OVA and GAPDH were performed using TaKaRa Ex Taq™, Hot-Start Version (Takara Bio, Inc). The primer sequences were as follows: GAPDH forward, 5′-GGTGAAGGTCGGTGGAACG-3′ and reverse, 5′-CTCGCTCCTGGAAGATGGTG-3′. The PCR conditions consisted of an initial denaturation step at 94 °C for 3 min, followed by 30 cycles of denaturation at 94 °C for 30 s, annealing at 64 °C for 30 s, and extension at 72 °C for 12 s.

### Transfections

HEK-293 T cells (3 × 10^5^ cells/well in 24-well plates) were transfected with 1.0 μg of the FrC-OVA-pAc-CAG-MCS2 and FrC-OVA-pVAX1-CAG-MCS plasmids using FuGENE-6 (Roche Diagnostics). At 20 h post transfection, RT-PCR was used to analyze the insertion of the FrC-OVA gene into the FrC-OVA-pAc-CAG-MCS2 and FrC-OVA-pVAX1-CAG-MCS plasmids. The details of the FrC-OVA RNA analysis are described in the previous section.

### Detection of the OVA protein in virus-infected or transfected cells by western blot analysis

HEK-293 T cells were infected at an MOI of 100 with rBV (1 × 10^8^ pfu) or transfected with FrC-OVA-pVAX1-CAG-MCS (1.0 μg) using FuGENE-6. At 24 h post infection, the cells were lysed with lysis buffer (50 mM Tris-HCl, pH 6.8, 0.1 M dithiothreitol, 2% SDS and 10% glycerol). The cell extracts were separated by SDS-PAGE, and proteins were blotted onto a PVDF membrane (Roche Molecular Diagnostics). The OVA protein was detected using an anti-OVA antibody (cat. no. SAB5300165; 1:2000 dilution; Sigma-Aldrich; Merck KGaA) and a horseradish peroxidase-conjugated anti-mouse IgG secondary antibody (1:10,000 dilution) using an ECL plus detection system (both GE Healthcare). GAPDH was used as an internal control.

### In vivo IFN-γ ELISPOT and CD8^+^ T-cell IFN-γ assays

FrC-OVA-pVAX1-CAG-MCS (100 μg), wtBV (1 × 10^8^ pfu), rBV (FrC-OVA-BV; 1 × 10^8^ pfu) or PBS was administered to the mice via intramuscular injection, and the injections were repeated on day 21. On day 35 post injection, the mice were sacrificed, and their spleens were isolated. Dissociation of the mouse spleens was performed using the gentleMACS Dissociator (Miltenyi Biotec GmbH) according to the manufacturer’s protocol. Following centrifugation (1500×g, 5 min, room temperature), the supernatant fluid was removed, and the pellet was resuspended in culture medium at the desired concentration. An aliquot of the suspended cells was used to assay the cell quantity, and the cell suspension was adjusted to a final density of 1xl0^7^ cells/ml. The splenocytes were used for the IFN-γ ELISPOT and CD8^+^ T-cell IFN-γ assays. IFN-γ release was measured using a commercial mouse IFN-γ ELISPOT Ready SET Go kit (eBioscience; Thermo Fisher Scientific, Inc.) according to the manufacturer’s instructions. Mouse spleen cells (2 × 10^6^ cells/well) were added to 96-well PVDF plates (Merck KGaA) that were precoated with a capture mouse anti-IFN-γ monoclonal antibody (5 μg/ml). The plates were treated with the OVA 257–264 peptide (GenScript Inc.) or HIV-1 Gag (#11057; 129 peptide consensus group M sequences, AIDS Research and Reference Reagent Program, Division of AIDS, National Institute of Allergy and Infectious Diseases, National Institutes of Health) at a concentration of 2.0 μg/well. After inoculation for 24 h at 37 °C, the plates were washed three times with PBS-Tween 0.05% and incubated with a biotinylated anti-IFN-γ monoclonal antibody at 100 μg/well for 2 h at room temperature. Following another round of washing, the plates were incubated with streptavidin-conjugated alkaline phosphatase for 1 h at room temperature. After the plates were washed with ELISPOT wash buffer, each well was incubated with AEC substrate solution (100 μl/well) for 24 h at 37 °C. The plate was air-dried overnight at room temperature in the dark. The ELISPOT plate was read by ZellNet Consulting, Inc.

The CD8^+^ T-cell IFN-γ assays were carried out using a commercial BD Cytofix/Cytoperm™ Plus Fixation/Permeabilization kit (BD Biosciences) according to the manufacturer’s protocols. The mouse spleen cells (2 × 10^6^ cells/well) were incubated with the OVA 257–264 peptide (2.0 μg/1.0 ml) and brefeldin A (1 μg/1.0 ml) for 4 h at 37 °C. Subsequently, the mouse spleen cells were incubated with a CD16/D32 monoclonal antibody (eBioscience; Thermo Fisher Scientific, Inc.) for 15 min on ice in the presence of a 2.4G2 monoclonal antibody to block FcγR binding. Following blocking, the cells were treated with FACS buffer (900 μl) and centrifuged at 2000×g for 5 min at 4 °C. The resuspended cells were treated with fixation/permeabilization solution (100 μl) for 20 min at 4 °C and then washed twice with BD Perm/Wash™ buffer. The cells were incubated with FITC-anti-mouse IFN-γ (10 μl; eBioscience; Thermo Fisher Scientific, Inc.) at 4 °C for 30 min in the dark and then washed twice with 1.0 ml BD Perm/Wash buffer. The plates were incubated with PE-conjugated anti-mouse CD8a (BD Biosciences) at 4 °C for 30 min in the dark and washed twice with 1.0 ml of FACS buffer. The cell pellets were suspended in FACS buffer (500 μl) and analyzed on a FACSCalibur instrument with CellQuest Pro software (BD Biosciences).

### In vivo immunization therapy against EG7-OVA cells

For prophylactic assessment, C57BL/6NCrSlc mice were immunized by a single intramuscular injection of FrC-OVA-pVAX1-CAG-MCS (100 μg), wtBV (1 × 10^8^ pfu), rBV (1 × 10^8^ pfu) or PBS. After 14 and 35 days, EG7-OVA cells (5 × 10^6^ cells/mouse) were transplanted subcutaneously into the immunized mice. The tumor volume was measured every 2 days for 3 weeks using a slide caliper and calculated according to the following formula: tumor volume (mm^3^) = 0.5 × length (mm) × width^2^ (mm^2^). To assess the therapeutic effect, C57BL/6NCrSlc mice were subcutaneously injected on day 0 with EG7-OVA cells (5 × 10^6^ cells/mouse). The survival rates of EG7-OVA-injected mice treated with rBV (1 × 10^8^ pfu), wtBV (1 × 10^8^ pfu), FrC-OVA-pVAX1-CAG-MCS (100 μg) or PBS (days 14 and 21) were evaluated.

### Statistical analysis

One-way analysis of variance followed by Tukey’s post hoc test or the Mann-Whitney U test were conducted for pairwise comparisons. All calculations were performed using the Statistica program (StatSoft, Inc.). The results are presented as median or mean values ± standard deviations. *P* < 0.05 was considered to indicate a statistically significant difference.

## Supplementary information

**Additional file 1: Figure S1.** RT-PCR analysis FrC-OVA RNA expression in Frc-OVA-pAc-CAG-MCS2 and FrC-OVA-pVAX1-CAG-MCS. (**a**, **c**) Schematic maps of Frc-OVA-pAc-CAG-MCS2 and FrC-OVA-pVAX1-CAG-MCS plasmids. RT-PCR amplification products analyzed by 2% agarose gel electrophoresis with ethidium bromide staining. RT-PCR analysis of FrC-OVA RNA was carried out using FrC-OVA specific primers with concomitant amplification of GAPDH mRNA. Lane 1: MOCK; lane 2: PC; lane 3: FrC-OVA RNA expression in FrC-OVA-pAc-CAG-MCS2 (**b**) and FrC-OVA-pVAX1-CAG-MCS (**d**).

## Data Availability

The datasets used and/or analyzed in the current study are available from the corresponding author upon reasonable request.

## Abbreviations

AcMNPV (wtBV): *Autographa californica* multiple nuclear polyhedrosis virus
CD8^+^T cells: CD8-positive T-lymphocyte
CMV promoter: Cytomegalovirus promoter
EG7-OVA cells: OVA-expressing EG7 lymphoma cells
ELISPOT: Enzyme-linked immunospot
FACS: Fluorescence-activated cell sorting
FrC: Fragment C
HEK-293 T cells: Human embryonic kidney cells 293
MHC: Major histocompatibility complex
OVA: Ovalbumin
rBV: Recombinant BV
Sf9 cells: *Spodoptera frugiperda* (Sf9) insect cells
