# Supplementary material for: Recombinant baculovirus expressing the FrC-OVA protein induces protective antitumor immunity in an EG7-OVA mouse model
Source: J Biol Eng. 2019 Oct 22;13:77. doi: 10.1186/s13036-019-0207-y (PMC6805443; doi:10.1186/s13036-019-0207-y)

Supplementary Data

Recombinant baculovirus expressing the FrC-OVA protein induces protective antitumor immunity in an EG7-OVA mouse model

Keigo Kondou, Tomoyuki Suzuki, Myint Oo Chang and Hiroshi Takaku

Figure S1. RT-PCR analysis FrC-OVA RNA expression in Frc-OVA-pAc-CAG-MCS2 and FrC-OVA-pVAX1-CAG-MCS. (**a,c**) Schematic maps of Frc-OVA-pAc-CAG-MCS2 and FrC-OVA-pVAX1-CAG-MCS plasmids. RT-PCR amplification products analyzed by 2% agarose gel electrophoresis with ethidium bromide staining. RT-PCR analysis of FrC-OVA RNA was carried out using FrC-OVA specific primers with concomitant amplification of GAPDH mRNA. Lane 1: MOCK; lane 2: PC; lane 3: FrC-OVA RNA expression in FrC-OVA-pAc-CAG-MCS2 (**b**) and FrC-OVA-pVAX1-CAG-MCS (**d**).

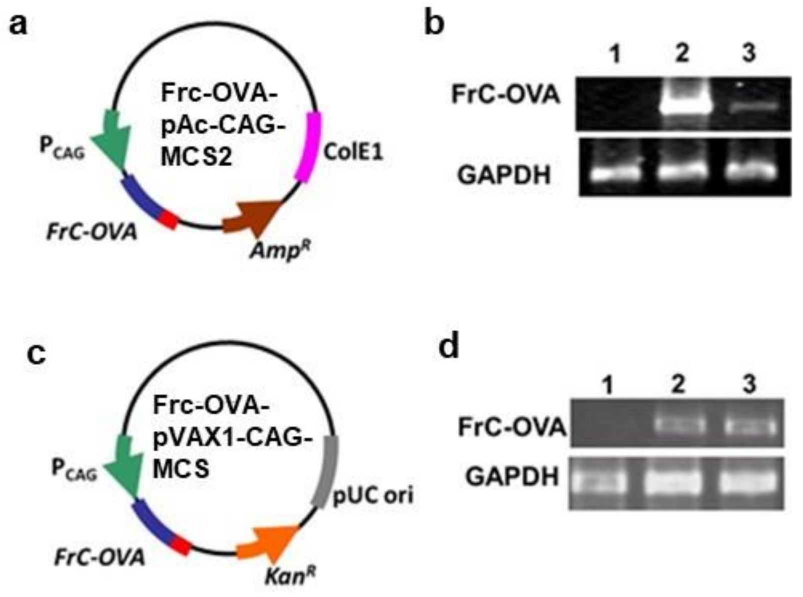

Supplement: Supplementary file 1 — Additional file 1: Figure S1. RT-PCR analysis FrC-OVA RNA expression in Frc-OVA-pAc-CAG-MCS2 and FrC-OVA-pVAX1-CAG-MCS. (a, c) Schematic maps of Frc-OVA-pAc-CAG-MCS2 and FrC-OVA-pVAX1-CAG-MCS plasmids. RT-PCR amplification products analyzed by 2% agarose gel electrophoresis with ethidium bromide staining. RT-PCR analysis of FrC-OVA RNA was carried out using FrC-OVA specific primers with concomitant amplification of GAPDH mRNA. Lane 1: MOCK; lane 2: PC; lane 3: FrC-OVA RNA expression in FrC-OVA-pAc-CAG-MCS2 (b) and FrC-OVA-pVAX1-CAG-MCS (d). [file 13036_2019_207_MOESM1_ESM.pdf]
